# Tuberculosis among Health-Care Workers in Low- and Middle-Income Countries: A Systematic Review

**DOI:** 10.1371/journal.pmed.0030494

**Published:** 2006-12-26

**Authors:** Rajnish Joshi, Arthur L Reingold, Dick Menzies, Madhukar Pai

**Affiliations:** 1 Division of Epidemiology, School of Public Health, University of California Berkeley, Berkeley, California, United States of America; 2 Department of Medicine, Mahatma Gandhi Institute of Medical Sciences, Sevagram, Maharashtra, India; 3 Montreal Chest Institute, McGill University, Montreal, Canada; BRAC, Bangladesh

## Abstract

**Background:**

The risk of transmission of Mycobacterium tuberculosis from patients to health-care workers (HCWs) is a neglected problem in many low- and middle-income countries (LMICs). Most health-care facilities in these countries lack resources to prevent nosocomial transmission of tuberculosis (TB).

**Methods and Findings:**

We conducted a systematic review to summarize the evidence on the incidence and prevalence of latent TB infection (LTBI) and disease among HCWs in LMICs, and to evaluate the impact of various preventive strategies that have been attempted. To identify relevant studies, we searched electronic databases and journals, and contacted experts in the field. We identified 42 articles, consisting of 51 studies, and extracted data on incidence, prevalence, and risk factors for LTBI and disease among HCWs. The prevalence of LTBI among HCWs was, on average, 54% (range 33% to 79%). Estimates of the annual risk of LTBI ranged from 0.5% to 14.3%, and the annual incidence of TB disease in HCWs ranged from 69 to 5,780 per 100,000. The attributable risk for TB disease in HCWs, compared to the risk in the general population, ranged from 25 to 5,361 per 100,000 per year. A higher risk of acquiring TB disease was associated with certain work locations (inpatient TB facility, laboratory, internal medicine, and emergency facilities) and occupational categories (radiology technicians, patient attendants, nurses, ward attendants, paramedics, and clinical officers).

**Conclusions:**

In summary, our review demonstrates that TB is a significant occupational problem among HCWs in LMICs. Available evidence reinforces the need to design and implement simple, effective, and affordable TB infection-control programs in health-care facilities in these countries.

## Abstract 2

A systematic review demonstrates that tuberculosis is an important occupational problem among health care workers in low and middle-income countries.

## Editors' Summary

**Background.**

One third of the world's population is infected with *Mycobacterium tuberculosis,* the bacterium that causes tuberculosis (TB). In many people, the bug causes no health problems—it remains latent. But about 10% of infected people develop active, potentially fatal TB, often in their lungs. People with active pulmonary TB readily spread the infection to other people, including health-care workers (HCWs), in small airborne droplets produced when they cough or sneeze. In high-income countries such as the US, guidelines are in place to minimize the transmission of TB in health-care facilities. Administrative controls (for example, standard treatment plans for people with suspected or confirmed TB) aim to reduce the exposure of HCWs to people with TB. Environmental controls (for example, the use of special isolation rooms) aim to prevent the spread and to reduce the concentration of infectious droplets in the air. Finally, respiratory-protection controls (for example, personal respirators for nursing staff) aim to reduce the risk of infection when exposure to M. tuberculosis is unavoidably high. Together, these three layers of control have reduced the incidence of TB in HCWs (the number who catch TB annually) in high-income countries.

**Why Was This Study Done?**

But what about low- and middle-income countries (LMICs) where more than 90% of the world's cases of TB occur? Here, there is little money available to implement even low-cost strategies to reduce TB transmission in health-care facilities—so how important an occupational disease is TB in HCWs in these countries? In this study, the researchers have systematically reviewed published papers to find out the incidence and prevalence (how many people in a population have a specific disease) of active TB and latent TB infections (LTBIs) in HCWs in LMICs. They have also investigated whether any of the preventative strategies used in high-income countries have been shown to reduce the TB burden in HCWs in poorer countries.

**What Did the Researchers Do and Find?**

To identify studies on TB transmission to HCWs in LMICs, the researchers searched electronic databases and journals, and also contacted experts on TB transmission. They then extracted and analyzed the relevant data on TB incidence, prevalence, risk factors, and control measures. Averaged-out over the 51 identified studies, 54% of HCWs had LTBI. In most of the studies, increasing age and duration of employment in health-care facilities, indicating a longer cumulative exposure to infection, was associated with a higher prevalence of LTBI. The same trend was seen in a subgroup of medical and nursing students. After accounting for the incidence of TB in the relevant general population, the excess incidence of TB in the different studies that was attributable to being a HCW ranged from 25 to 5,361 cases per 100, 000 people per year. In addition, a higher risk of acquiring TB was associated with working in specific locations (for example, inpatient TB facilities or diagnostic laboratories) and with specific occupations, including nurses and radiology attendants; most of the health-care facilities examined in the published studies had no specific TB infection-control programs in place.

**What Do These Findings Mean?**

As with all systematic reviews, the accuracy of these findings may be limited by some aspects of the original studies, such as how the incidence of LTBI was measured. In addition, the possibility that the researchers missed some relevant published studies, or that only studies where there was a high incidence of TB in HCWs were published, may also affect the findings of this study. Nevertheless, they suggest that TB is an important occupational disease in HCWs in LMICs and that the HCWs most at risk of TB are those exposed to the most patients with TB. Reduction of that risk should be a high priority because occupational TB leads to the loss of essential, skilled HCWs. Unfortunately, there are few data available to indicate how this should be done. Thus, the researchers conclude, well-designed field studies are urgently needed to evaluate whether the TB-control measures that have reduced TB transmission to HCWs in high-income countries will work and be affordable in LMICs.

**Additional Information.**

Please access these Web sites via the online version of this summary at http://dx.doi.org/10.1371/journal.pmed.0030494.

• US National Institute of Allergy and Infectious Diseases patient fact sheet on tuberculosis

• US Centers for Disease Control and Prevention information for patients and professionals on tuberculosis

• MedlinePlus encyclopedia entry on tuberculosis

• NHS Direct Online, from the UK National Health Service, patient information on tuberculosis

• US National Institute for Occupational Health and Safety, information about tuberculosis for health-care workers

• American Lung Association information on tuberculosis and health-care workers

## Introduction

The risk of transmission of Mycobacterium tuberculosis from individuals with tuberculosis (TB) to other patients and to health-care workers (HCWs) has been recognized for many years [1,2]. This risk is greater when larger numbers of infectious (smear-positive) TB patients are managed at a health-care facility, and can be reduced with implementation of effective infection-control measures [3,4]. In the United States and other high-income countries, the risk of nosocomial transmission of TB was high in the pre-chemotherapy era, but declined with the reduction in incidence of TB disease in the population [3]. This trend, however, changed between 1985 and 1993, when several outbreaks of multidrug-resistant tuberculosis (MDR-TB) were reported in nosocomial and congregate settings in the United States [5,6]. This led to recommendations for a comprehensive set of infection-control practices to protect HCWs and reduce nosocomial transmission [1,4]. In the years following the publication of these recommendations, there was a dramatic decline in the burden of TB among HCWs [1,4,7]. In a recent review of TB among HCWs in high-income countries, the overall incidence of TB disease in the general population and native-born HCWs was less than 10 and 25 per 100,000 per year, respectively [8].

The situation is very different in low- and middle-income countries (LMICs), which account for more than 90% of the global TB burden [9,10]. Because these countries have high TB rates and limited resources [11,12], they focus largely on case detection and treatment using the DOTS strategy [1,9]. In these countries, even low-cost strategies to reduce TB transmission in health-care facilities are seldom implemented [3,13].

We conducted a systematic review to summarize the evidence on the incidence and prevalence of latent TB infection (LTBI) and TB disease among HCWs in LMICs. We specifically addressed the following questions: (1) What is the prevalence of LTBI and what are the risk factors for LTBI in HCWs? (2) What is the incidence of LTBI in HCWs and what risk factors are associated with higher incidence rates? (3) What is the incidence of TB disease in HCWs and how does it compare with the incidence in the population? (4) Are certain occupations, or some work locations within a health-care facility, at higher risk of TB than others? (5) How effective are various strategies in reducing the incidence of LTBI and/or disease among HCWs in LMICs?

## Methods

### Search Strategy

We searched the following electronic databases for primary studies: PubMed (http://www.ncbi.nlm.nih.gov/entrez/query.fcgi?DB=pubmed, 1950 to December 2005), BIOSIS (http://scientific.thomson.com/products/bp, 1969 to December 2005), Embase (http://www.embase.com, 1974 to November 2005), and Web of Science (http://isiknowledge.com, 1945 to December 2005). Our search strategy included terms such as “tuberculosis,” “health personnel,” “health-care worker,” “nosocomial,” “infection control,” “disease transmission,” “occupational exposure,” and “nosocomial tuberculosis.” (Details of the complete search strategy are provided in Table S1.) We hand-searched the indices of the *International Journal of Tuberculosis and Lung Disease; Tuberculosis;* and *Tubercle and Lung Disease* for relevant articles not already captured by the electronic searches. We identified additional studies by contacting experts in the field and by searching reference lists of primary studies, review articles, and textbook chapters.

### Study Selection

Our search strategy aimed to identify all the available published studies in the English language that reported data on the incidence and prevalence of LTBI and TB disease in HCWs. Although non-English studies were excluded, we extracted data from studies that had English abstracts, and these limited data are included in Tables S1 and S2. This included cross-sectional tuberculin surveys, cohort studies on tuberculin-conversion rates, retrospective or prospective studies on the incidence of TB disease, studies on risk factors for acquiring LTBI or TB disease, and studies documenting the effect of preventive strategies aimed at reducing nosocomial transmission. We restricted the review to studies conducted in countries classified by the World Bank as low or middle income [14]. We excluded case reports or case series of nosocomial transmission or outbreaks, as well as conference abstracts. Two reviewers (RJ and MP) independently screened the citations (titles and abstracts) identified from all sources. Subsequently, full-text articles of the studies selected in the initial screen of titles and abstracts were reviewed to identify the final set of eligible studies.

### Data Extraction

Two reviewers (RJ and MP) independently extracted data from a subset of eligible studies. The inter-rater agreement on TB outcomes (such as LTBI incidence and prevalence, and TB disease incidence, etc.) was 100% in this pilot study. Subsequently, data from the full set of included studies were extracted by one reviewer (RJ). Data extracted included: country, survey year, type of health-care facility, number of TB patients managed in the facility, infection-control practices (such as personal protection, administrative measures, engineering controls, etc.) in the facility at the time of the study, prevalence and incidence of LTBI, prevalence and incidence of TB disease, risk factors for LTBI or TB disease, infection-control interventions (personal, administrative, and engineering controls), and evaluations of their effectiveness, delays in diagnosis at the facility, and demographic and other relevant details about HCWs included in the studies. We used the following definitions to standardize the data-extraction process.

Health-care facility: All facilities where patients seek health care, including hospitals, clinics, dispensaries, health centers, and imaging and laboratory facilities. We did not include prisons, nursing homes, correctional facilities, and other congregate settings.

HCW: Any individual who works in a health-care setting including, but not restricted to, physicians, nurses, allied health personnel (nursing assistants, operation theater technicians, etc.), health educators, social workers, midwives, community health workers based in hospitals, laboratory personnel, pharmacists, radiographers, volunteers, orderlies, and health-facility administrators.

LTBI: A positive tuberculin skin test (TST) done by any standard method using 1TU (tuberculin unit) or 2TU of purified protein derivative of tuberculin (PPD) RT23 or 5TU of PPD-S, with induration size ≥ 10 mm on a single test [15]. Owing to the high prevalence of BCG vaccination and non-tuberculous mycobacteria (NTM) in LMICs [16], a positive result after the second TST in a two-step TST (i.e., boosting) was excluded from the calculation of LTBI prevalence.

Tuberculin conversion: Defined as a newly positive TST after a documented negative-baseline TST (at any time after a negative two-step baseline, or more than 1 y after a negative single TST). An increase of 10 mm over the baseline was defined as conversion [4].

TB disease: Included all forms of pulmonary, as well as extra-pulmonary, TB where a definitive (microbiologically confirmed), or presumptive (based on clinical, imaging, or pathology criteria) diagnosis was made. The definition included self-reported past treatment for TB disease.

Income category definitions: The countries were grouped according to 2004 gross national income per capita criteria as suggested by the World Bank, which classifies LMICs as those with per capita income value of less than US$10,066 [14].

Infection-control interventions were defined as any personal protection (including, but not limited to, respirators), administrative measures (including, but not limited to, early diagnosis and isolation policy, reducing time for which TB patients would be hospitalized, and reducing waiting times for infectious patients in outpatient and radiology facilities), and environmental controls (including, but not limited to, negative-pressure isolation rooms, HEPA filters, etc.)

Data on the estimated incidence of all forms of TB disease in the general population were obtained from the World Health Organization's (WHO) global TB database [17]. All risk estimates among HCWs were calculated with respect to these country incidence rates. Since there may be considerable variations in TB incidence rates within a specific country, we also used the authors' estimates of regional/local incidence rates, if reported.

### Data Synthesis

Studies were heterogeneous in many respects, including baseline TB incidence in the population, institutional TB case loads, types of tests used to detect TB, job descriptions and classifications of HCWs, and preventive measures used at health-care facilities. Hence we analyzed the studies in prespecified subgroups. Studies of medical or nursing students were analyzed separately, as their risk of exposure may be different from other HCWs. Studies on TB disease were analyzed separately from studies on LTBI.

The incidence rates of LTBI and TB disease among HCWs, and corresponding estimates in the general population, were used to determine the excess risk among HCWs attributable to nosocomial exposure. We calculated the risk estimates for the incidence of TB disease in HCWs for various occupations and work locations, with incidence of TB disease in the general population as a reference. Data were analyzed using Stata (Version 9) and Meta-DiSc (Version 1.2) software.

In meta-analyses, heterogeneity refers to a substantial degree of variability in study results. Such heterogeneity can be due to differences in methodological quality, study design, sampling variability, and study populations across studies. In the presence of significant heterogeneity, pooled or summary estimates from meta-analyses are difficult to interpret. We addressed heterogeneity using subgroup (stratified) analyses. Because the studies estimating the prevalence of LTBI had comparable methodologies, we generated pooled (summary) estimates by using a fixed-effects model, where studies were weighed by the overall sample size, and we corrected for over-dispersion to allow for heterogeneity that was due to between-study variability.

## Results

### Description of Included Studies

**Figure 1:**
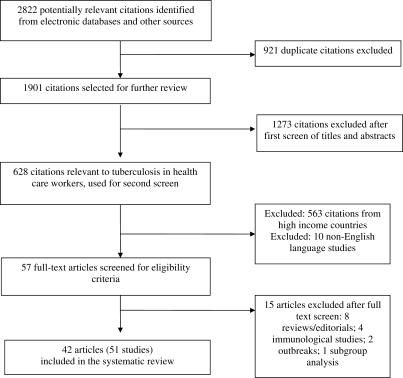
Study Flow Chart

Of the 1,901 unique citations identified in the literature search, 42 articles describing the results of 51 studies met our eligibility criteria (Figure 1). The prevalence of LTBI was determined by tuberculin surveys in 18 studies (35.3%) [18–35]. Seven studies (11%) [18–22,36,37] determined the incidence of LTBI using tuberculin conversions. Twenty (39.2%) studies [23,38–56] determined the incidence of TB disease in HCWs. Six studies (11.7%) [21,22,24,44,48,57] reported the use of infection-control measures to reduce nosocomial transmission. Environmental attributes such as type of ventilation in the health-care facility, area devoted to patient care, patient and HCW crowding, and cumulative hospital stay of infectious patients were not reported by any study.

### Prevalence of LTBI and Risk Factors for LTBI

#### Medical and nursing students.

**Figure 2:**
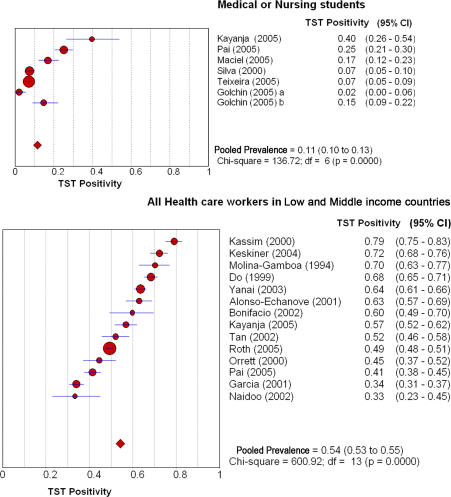
Prevalence of TB Infection in HCWs as Determined by TST Surveys The circles and the lines represent the point estimates and 95% CIs, respectively. The size of the circle indicates the study size, and the diamond indicates the pooled estimate (weighted average) and its 95% CI. The heterogeneity chi-square statistics are 142.6 (*p* < 0.01) for the medical students and 600.9 (*p* < 0.01), for all HCWs, indicating significant heterogeneity across studies. (a) Indicates estimate of TST positivity in nursing students at entry into nursing school. (b) Indicates estimate of TST positivity after 3 y in nursing school.

As seen in Figure 2 and Table 1, the prevalence of LTBI in medical or nursing students varied widely from 2% (Iran [25]) to 40% (Uganda [26]). The prevalence estimates correlated well (correlation coefficient *[R]* = 0.91, *p* = 0.01) with TB disease incidence rates in the general population (e.g., ranging from 28 per 100,000 in Iran to 403 per 100,000 in Uganda).As shown in Table 1, levels of training and age were associated with the prevalence of LTBI in most studies. The prevalence of LTBI in senior years was two to three times higher compared with junior years in two studies from Brazil [24,27]. A study from India [28] reported a 4-fold higher prevalence in medical students who were more than 23 y of age than in medical students aged 18–20 y (corresponding to an additional 3–5 y spent in training). The overall pooled prevalence of LTBI was 12% (95% confidence interval [CI] 10 to 13) among medical or nursing students, excluding the study by Levy et al. [20], where all medical students had received BCG vaccination within the previous 6 mo, and 70% of them were TST-positive. In other studies, where BCG was usually given in childhood, the presence of a BCG scar was not significantly associated with LTBI.

**Table 1:**
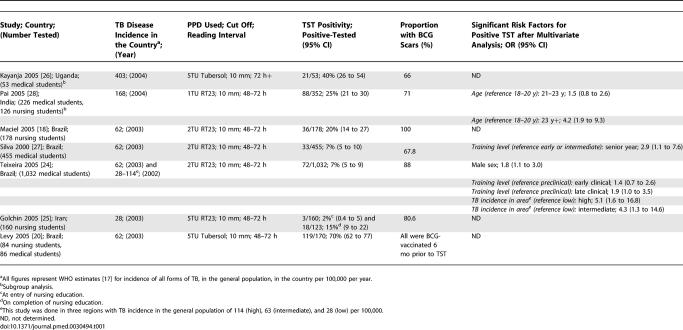
Prevalence of LTBI in Medical or Nursing Students as Determined by TST Surveys in LMICs

#### All HCWs.

As shown in Figure 2 and Table 2, the prevalence of LTBI in all HCWs ranged from 33% (95% CI 23 to 45) [29] to 79% (95% CI 75 to 82) [30] in various studies, with a pooled prevalence estimate of 54% (95% CI 53 to 55). Increasing age and duration of employment in the health-care facility (indicating longer cumulative exposure), were associated with higher prevalence of LTBI in most studies (Table 2). The prevalence of LTBI in HCWs increased by 1.04 times (95% CI 1.02 to 1.07) with each additional year of increase in age [26,31], and by 1.5 (95% CI 1.0 to 2.2) [23] to 2.4 (95% CI 1.1 to 5.0) [31] times with employment duration of more than 1 y. The prevalence of LTBI was 3-fold higher [28] with ≥10 y of employment. Working in medical wards, participation in procedures such as sputum collection and autopsies, and a history of contact with TB patients were independent occupational risk factors for LTBI.

**Table 2:**
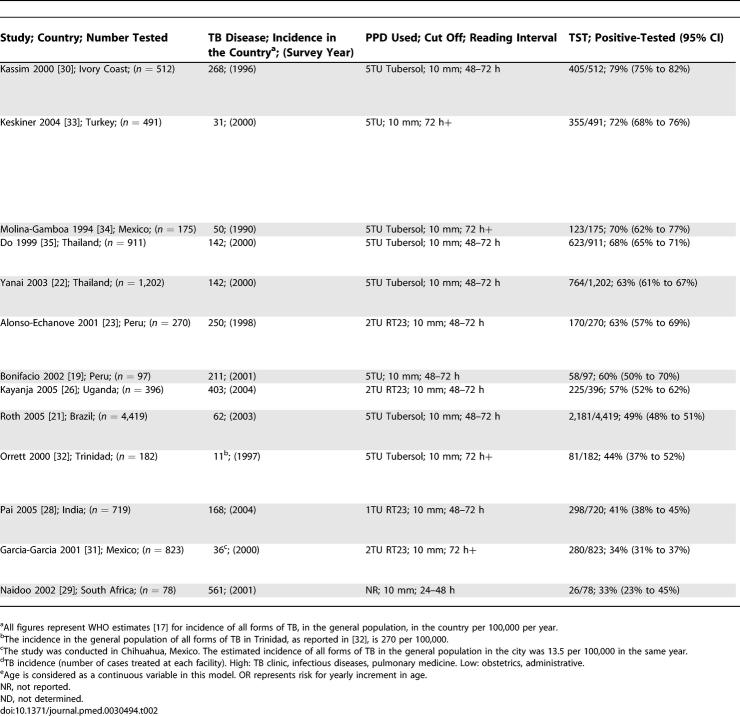
Prevalence of LTBI in All HCWs as Determined by TST Surveys

**Table 2:**
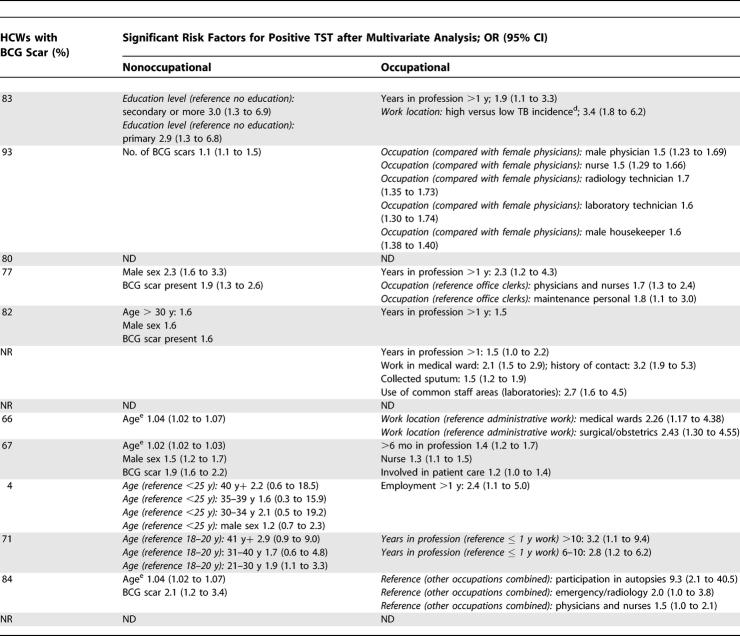
Extended.

Several studies reported a prevalence of LTBI in nurses, a subgroup with a high level of patient contact, and thus potential exposure to TB cases. As seen in Table 3, the prevalence of LTBI among nurses ranged from 43% to 87%. It was reported in eight studies that the prevalence of LTBI in nurses was higher than that in other HCWs (ranging from being 1.3% higher [22] to 35.6% higher [31]. One study, however [32], reported a lower prevalence in nurses, compared to other HCWs).

**Table 3:**
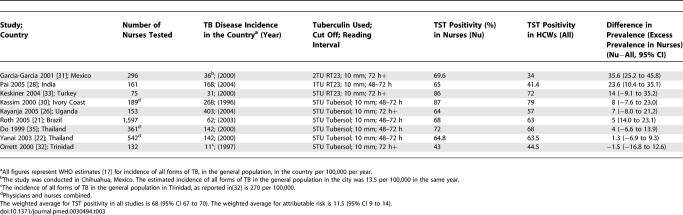
Prevalence of LTBI in Nurses

### Incidence of LTBI (Tuberculin Conversion) and Risk Factors

In six out of the seven studies covered in Table 4, the annual risk of TB infection ranged from 3.9% to 14.3%, after accounting for the incidence of TB infection in the general population, and the risk attributable to occupational exposure ranged from 2.6% to 11.3%. (A study in medical students by Levy et al. [20], where all participants were BCG-vaccinated 6 mo prior to the first TST was excluded, as this vaccination program could be responsible for a low LTBI incidence of 0.5% in this study). There was marked, although non-significant, correlation between the incidence of LTBI and TB hospital admissions per year (*R* = 0.86, *p* = 0.13). A higher level of clinical training (odds ratio [OR] = 4.77, 95% CI 1.01 to 22.46) [36], BCG vaccination after baseline TST (OR 2.9, 95% CI 1.1 to 7.6) [21], nursing occupation (OR 1.7, 95% CI 1.1 to 2.7) [21], and recent exposure to TB (OR 1.6, 95% CI 1.0 to 2.6) [21] were independent risk factors for TST conversion in these studies.

**Table 4:**
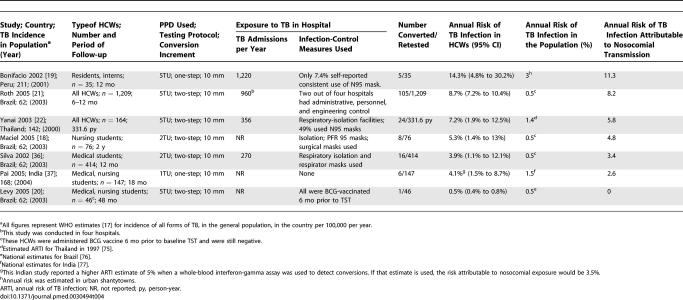
Incidence of LTBI or Tuberculin Conversion in HCWs

### Incidence of TB Disease

Results of studies that estimated the incidence of TB disease are shown in Table 5. Two studies (one from Russia [41] and another from South Africa [39]) reported lower TB disease incidence in HCWs than in the general population. In South Africa, the incidence of TB in the general population increased rapidly (from 321 per 100,000 per year in 1991 to 1,250 per 100,000 per year in 1996), with 44% of these cases being attributed to HIV infection [54]. The incidence of TB disease in Russia in the general population (113 per 100,000) was much higher than in the Samara Oblast (74.9 per 100,000), where a study by Dimitrova et al. [41] was carried out, which could be responsible for the low attributable risk estimates in the Dimitrova study [41]. In the remaining studies, the risk to HCWs of TB disease attributable to nosocomial exposure ranged from 25 to 5,361 per 100,000 per year. HCWs in facilities with fewer HCWs for every TB patient seen (HCW-to-patient ratio less than 50 per 100 TB patients), had a higher incidence of TB disease in HCWs (*R* = −0.45, *p* = 0.18).

**Table 5:**
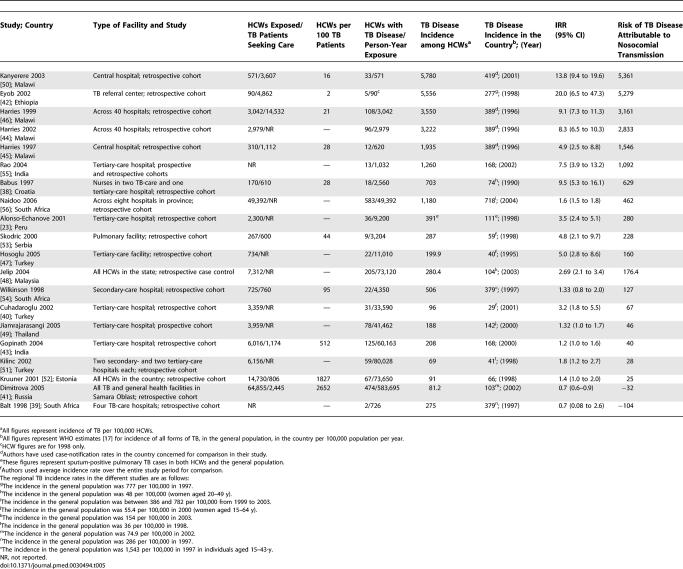
Incidence of TB Disease (All Forms) in HCWs

### Association between Work Location and Job Categories with Risk of TB Disease

Studies that reported rates of TB disease by work location and occupational categories are shown in Table 6. We calculated incidence rate ratios (IRRs) by using an estimated general population incidence rate for all TB cases in the country as a comparison. Workers in TB inpatient facilities (IRR ranged from 14.6 to 99.0), laboratories (IRR = 78.6), general medicine wards (IRR ranged from 3.9 to 36.6), and emergency rooms (IRR ranged from 26.6 to 31.9) had a higher risk for TB disease compared with the general population. Workers in outpatient medical facilities had an intermediate risk (IRR ranged from 4.2 to 11.6), and workers in surgery, obstetrics and gynecology, administration, and operating theaters had a lower risk. There was considerable heterogeneity in the risk of TB disease between different occupations: radiology technicians, patient attendants, nurses, ward attendants, paramedics, clinical officers, laboratory personnel, and physicians had a high incidence of TB disease, while the incidence of TB disease was lowest in administrative staff.

**Table 6:**
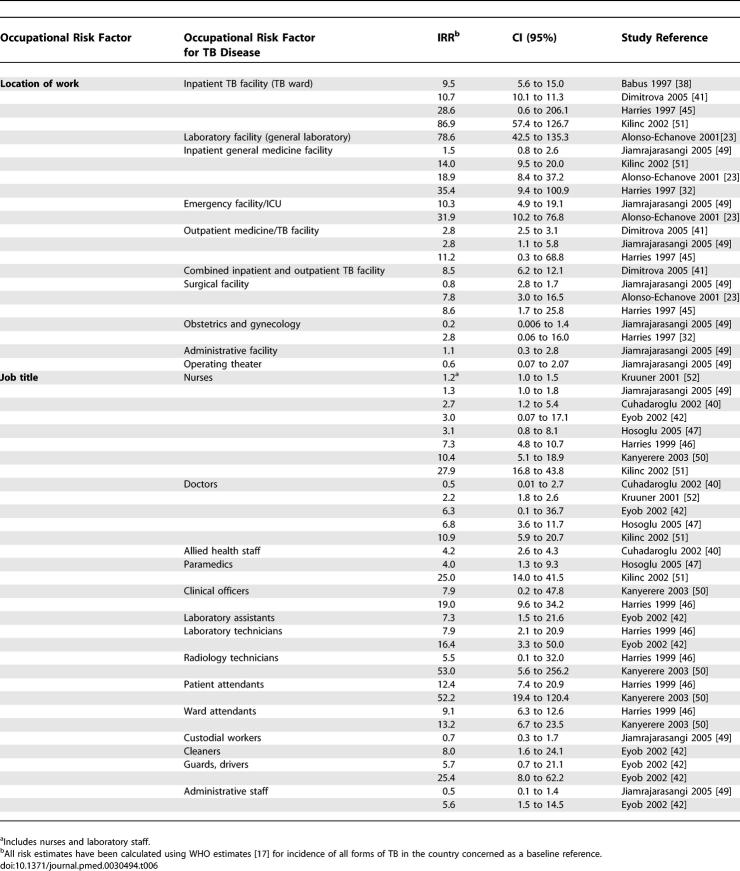
Occupational Risk Factors for TB Disease in HCWs

### Impact of Infection-Control Strategies on the Incidence of TB Infection or Disease

Most authors reported that no specific TB infection-control programs were being used in the health-care facilities where the studies were carried out. Only three studies [21,22,44] evaluated the impact of multiple infection-control strategies on the risk of TB infection or disease. Another two studies [24,48]analyzed whether a lack of personal-protection measures was associated with a risk of TB infection. One study [57] evaluated the knowledge, attitudes, and use of TB infection-control measures by HCWs.

As seen in Table 7, Harries at al. [44] evaluated the impact of multiple administrative control measures which were implemented in 40 district and mission hospitals in Malawi, following adoption of infection-control guidelines. The data were collected by interviewing HCWs and by screening the TB registers at these facilities. The study revealed that the infection-control guidelines were not uniformly implemented, and the median compliance with various measures was 76% (range 3% to 100%). There was a non-significant decrease in TB disease incidence after 1 y of implementing these measures.

**Table 7:**
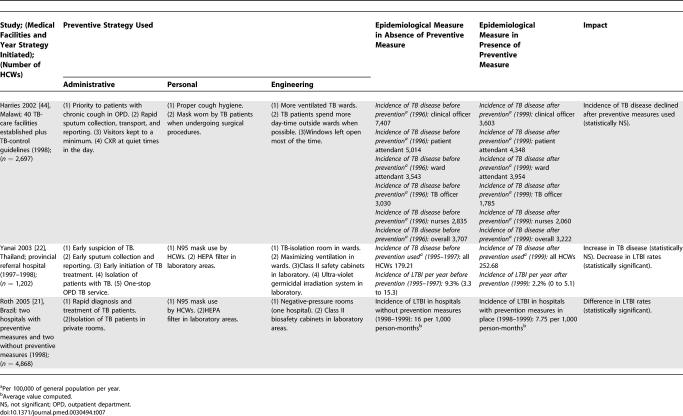
Impact of Multiple Administrative, Personal, and Engineering Control Measures on Nosocomial Transmission of TB

The introduction of multiple administrative, personal, and engineering controls in a single hospital in Thailand [22] resulted in a significant drop in the annual incidence of LTBI in HCWs from 9.3% to 2.2%. However, the incidence of TB disease in HCWs showed a non-significant increase (from 179 to 252 per 100,000) 1–2 y after initiation of these control measures. During the course of this study [22], the proportion of HIV-positive TB patients treated at this facility increased from 3% to 57%; if there was a similar increase in HIV among HCWs, the incidence could have increased despite a fall in new infections**.**

In another study from Brazil [21], a cross-sectional tuberculin survey determined the baseline LTBI prevalence in four hospitals. Hospital A initiated administrative controls and provided N95 respirators for all HCWs required to enter a TB-isolation room. Hospital B had initiated administrative controls 3 mo before the baseline TST testing and, at the onset of the study, had introduced N95 respirators and had began construction of negative-pressure isolation rooms. Hospitals C and D had no TB-control measures in place throughout the study. Baseline TST positivity was significantly different in the four hospitals (46.7%, 69.6%, 65.8%, and 62.2% in hospitals A, B, C, and D, respectively). After 1 y, the incidence of LTBI (in initially tuberculin-negative workers) was significantly lower in hospitals A and B, which had implemented multiple infection-control measures, compared with the other two hospitals.

In a case-control study by Jelip et al. [48], HCWs with TB disease were 5.9 times (95% CI 0.76 to 46.4) more likely to have poor knowledge about TB transmission, and 4.3 times (95% CI 0.95 to 19.8) more likely to be unaware of the need for respiratory protection. In a study among medical students [24], although 90% were aware of the risk of TB transmission, only 46% reported the use of personal-protection measures. In a study from Thailand [57], although 97% of HCWs were aware of TB infection-control policies, only 52% used personal-protection measures (e.g., respirators), and only 72% implemented respiratory isolation for TB cases. Failure to use personal protection was associated with a 2.6-fold (95% CI 1.06 to 6.64) increased risk of TB disease in HCWs [46].

## Discussion

### Principal Findings

Our systematic review of 51 studies demonstrates that the prevalence (range 33% to 79%) and incidence (range 0.5% to 14.3% per year) of LTBI, and the attributable risk of TB disease due to nosocomial exposure (from 25 to 5,361 per 100,000 per year), were high among HCWs in LMICs. The attributable risk was higher in health-care facilities that had more TB patients per HCW. Certain work locations (inpatient TB facility, laboratory, general medicine, and emergency facilities) and occupational categories (radiology technicians, patient attendants, nurses, ward attendants, paramedics, and clinical officers) were associated with a higher risk of TB disease. Finally, there is little published evidence on the effects of infection-control measures in LMIC.

**Figure 3:**
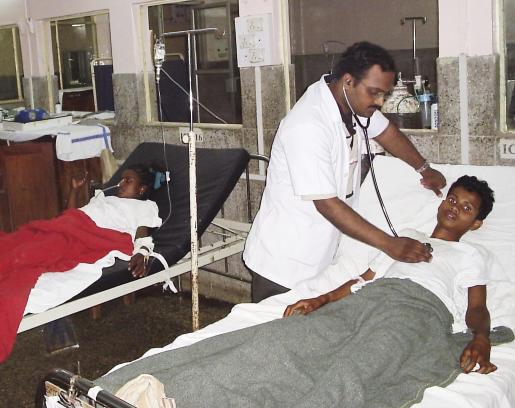
A Young Medical Trainee Examines a Patient with Pulmonary TB at a Rural Hospital in India In such low-income countries, more years of clinical training and greater exposure to TB patients are important risk factors for acquiring new TB infection.

In a recent review of TB among HCWs in high-income settings, the prevalence of LTBI ranged from 5% to 55%, in different occupations [8]. The annual risk of TB infection ranged from 0.1% to 10% in studies published prior to 1995 [56], but only from 0.1% to 1.2% after the widespread introduction of infection-control measures [8]. The annual incidence of TB disease in the general population was much lower (2–55 per 100,000) in HCWs in high-income countries (excluding foreign-born HCWs) [8], than among HCWs in LMICs (69–5,780 per 100,000). Health-care facilities in LMICs had a median of 36 (range 2–2,652) HCWs per 100 TB patients treated at the facility, which is much lower than facilities in high-income countries, which have a median of 6,450 (range 100–77,000) HCWs per 100 TB patients [58]. Thus, HCWs in low-income countries are likely to experience significantly higher TB exposure (Figure 3), and it is therefore not surprising that the epidemiology of TB among HCWs is very different in high-income countries versus LMICs.

### Strengths and Limitations of the Review

Our systematic review had several strengths. We used a comprehensive search strategy using multiple sources and databases to retrieve relevant studies. Two reviewers (RJ and MP) independently selected and extracted data from the included studies. Subgroup analyses were done to minimize heterogeneity across studies. Data were pooled only when studies were reasonably consistent in their methods. However, our review had certain limitations. First, despite the comprehensive literature search, a few eligible studies were missed, because we included only English-language studies. Our literature search had identified ten non-English articles [59–68] which potentially could have been included in the final review. Of these ten papers, seven provided an abstract in English, and the summary results are presented in Table S2. As seen in Table S2, apart from one study [63], the overall results are fairly similar to the English studies included in our review. This finding, although reassuring, does not completely rule out a language bias in our review. Second, publication bias is a known problem in systematic reviews and meta-analyses. If studies with high TB rates among HCWs were more likely to have been published, this may over-estimate the TB burden among HCWs. Thirdly, there were limited data on the magnitude of TB exposure in HCWs. Most studies did not report even simple indicators of exposure, such as the number of TB patients cared for at the facility, the number of inpatient days of TB patients, or the number of TB patients per HCW. Hospital infection-control policies and programs are important, yet often unmeasured, potential confounders—and these were not described in most studies. Finally, although we pooled prevalence estimates because of methodological similarity between studies on LTBI prevalence, the pooled averages will need to be interpreted with caution because of heterogeneity in study results.

### Limitations of Primary Studies Included in the Review

In addition to the limitations of the review, the primary studies included within its scope had several limitations. These limitations are discussed separately for each of the four main outcomes in our review: (1) the prevalence of LTBI; (2) the incidence of LTBI; (3) the incidence of TB disease; and (4) the impact of infection-control measures.

### Prevalence of LTBI in HCWs

Studies that reported LTBI prevalence among HCWs had several limitations. The first limitation pertains to the use of the TST. The prevalence of occupational LTBI could have been over-estimated because it was based on TST. The TST detects lifetime cumulative occupational plus nonoccupational exposure to *M. tuberculosis,* as well as the effects of NTM exposure and BCG vaccination. Prevalence of NTM and its effect on TST is difficult to estimate because studies from several countries were included, and there are no data on NTM prevalence in each setting.

The practice, timing, and frequency of BCG vaccination vary widely across countries, which complicates the analyses as BCG can be an important cause of false-positive TST. The results of TST are also influenced by the type of test material (PPD), technique of reading, and definition of a positive test. Although all studies used the definition of 10 mm or more induration after 48–72 h for TST to be positive, the PPD used varied, which could reduce comparability of studies. These limitations may affect the prevalence of LTBI, but should not affect the analysis of risk factors associated with LTBI.

Recently, interferon-gamma release assays (IGRAs) have become available for the diagnosis of LTBI. In contrast to the TST, IGRAs use antigens that are significantly more specific than PPD. Thus, IGRAs are highly specific and are therefore less likely to be affected by previous BCG vaccination and NTM exposure [69,70]. Only one study in our review used an IGRA for estimation of prevalence among HCWs. This study showed comparable LTBI prevalence using TST and IGRA, and high agreement between the test results [28].

Another major limitation of prevalence studies is the lack of concurrent data on LTBI prevalence in the population. Thus, it is not easy to determine whether HCWs had a significantly higher LTBI prevalence than the community. However, despite this limitation, our review shows that the prevalence of LTBI was lower among young medical or nursing students newly entering the health-care profession, but increased with each year of training (an index of cumulative exposure). Similarly, the prevalence of LTBI among other HCWs increased with duration of employment, again reflecting cumulative exposure. HCWs whose occupation involves closer patient contact (such as nurses) also had higher LTBI prevalence. These results indirectly suggest that nosocomial TB contributes to the burden of LTBI among HCWs.

### Incidence of LTBI in HCWs

Almost all studies that estimated the incidence of new TB infections used serial tuberculin skin testing. In addition to the known limitations of TST, serial TST has additional problems such as boosting, choice between a single-step or a two-step baseline protocol, and the definitions used for conversion. Most studies followed the two-step testing protocol so as not to overestimate true LTBI incidence due to boosting. Only one study used an IGRA for estimating the rate of new TB infection [37]. This study found a higher conversion rate when an IGRA was used, raising the possibility that IGRAs may be more sensitive for recent infection than the TST [37]. This hypothesis deserves further study.

Despite the above limitations, the results suggest that HCWs have a higher risk of TB infection than the estimates of risk in the general population. The high attributable risk estimates for LTBI incidence provide the most convincing evidence for nosocomial transmission of TB in health-care settings. In these studies, more years of clinical training and greater exposure to TB patients were risk factors for new infection, and this provides additional support for nosocomial transmission.

### Incidence of TB Disease in HCWs

The incidence of TB disease in HCWs was generally higher than the estimated TB rates in the general population. However, several methodological problems may affect the interpretation of these studies. HCWs may be more likely to seek medical care, and hence case-detection rates may be higher than in the general population. Our review included probable and self-reported TB cases, which also could have inflated the incidence of TB disease among HCWs. On the other hand, HCWs are less likely to develop TB because HCWs have a higher average socio-economic status, and are younger and healthier, than the general population in LMIC (i.e., healthy-worker effect) [71]. We used WHO estimates for the incidence of TB in the relevant country to ensure comparability of results across studies. Using data from a single source ensures uniformity in determining attributable risk for different studies. However, there may be substantial regional variation of incidence within countries, and it would seem that regional estimates of TB incidence would be more valid to compare with rates of TB in a particular institution. However, the estimates of relative and attributable risk were not markedly different when we analyzed the studies using WHO data, or using local estimates provided by the authors of the study concerned.

Despite the above limitations, most studies reported higher estimates of TB disease among HCWs than in the general population, and this is suggestive of nosocomial transmission. The high rates of TB disease among young HCWs are particularly worrisome. Some of this may be explained by coinfection with HIV—particularly in countries with a very high prevalence of HIV, such as in Sub-Saharan Africa. Few studies reported the prevalence of HIV infection among HCWs,and thus the impact of HIV on TB disease among HCWs could not be addressed.

Molecular studies involving DNA fingerprinting could provide confirmatory evidence of nosocomial transmission, as they have in high-income countries [72]. However, our literature search revealed only a single case report [73] from a LMIC in which molecular methods were used to confirm nosocomial transmission of M. tuberculosis to a nurse.

### Impact of Infection-Control Measures on TB in HCWs

Only three studies [21,22,44] in this review assessed the impact of infection-control measures on reducing the risk of TB in HCWs. Thus, it is difficult to make any inferences regarding effectiveness of control measures. One study determined that administrative measures had little impact on the development of TB disease, but the study considered a period of only 1 y—a relatively short interval to detect changes in this outcome [44]. Two studies that measured the incidence of TB infection detected significant reduction within 1 y following the introduction of multiple infection-control measures [22,31]. In one of these studies [22], the incidence of active disease in HCWs actually increased over the study period, but this could have been due to an increasing HIV prevalence in HCWs, which was not measured. The other study [31] compared TB incidence rates in hospitals with different infection-control policies, and other differences identified between the hospitals could have confounded the estimates. Taken together, the limited available evidence suggests that a reduction in the risk of TB infection is possible with simple administrative controls, but this needs to be evaluated in larger, better-controlled studies.

In summary, there is consistent epidemiologic evidence that TB is an important occupational disease in HCWs. There is clear evidence of heavy exposure, with little or no infection-control measures in place. Thus, it is not surprising that there is consistent evidence of excess prevalence and incidence of TB infection, as well as a higher incidence of TB disease among HCWs than in the general populations in the same LMICs. Although estimates vary widely, infection and disease are roughly correlated with indicators of exposure—including more years of work, or clinical training, and work that has been identified as high risk among HCWs in high-income countries. Finally, there is evidence, albeit limited and weak, that the incidence of infection drops after the implementation of infection-control measures. This epidemiological evidence implies that a substantial proportion of LTBI and TB disease in the HCWs in LMICs is the result of nosocomial TB transmission.

### Conclusions

Our review presents fairly strong evidence that nosocomial TB is an important occupational problem among HCWs in LMICs, and reduction of that risk should be a priority. Currently available evidence is limited, but it suggests that relatively simple interventions, such as early diagnosis of TB, segregation of infectious TB patients, or education and training of HCWs, might be effective. Additional low-cost measures could include engineering controls such as exhaust ventilation, improved natural ventilation, or sunlight [74]. However, well-designed field studies evaluating the cost, feasibility, and effectiveness of these interventions in resource-limited settings are urgently needed.

There are several important reasons as to why nosocomial transmission of TB should be addressed in LMICs. First, occupational TB can lead to the loss of skilled workers, and this can adversely impact health-care services in the long run. Second, transmission of TB can have serious, and even fatal, consequences for patients and HCWs. This is particularly true with MDR-TB strains, and in patient populations with high HIV seroprevalence. Hospitals have been shown to be important focal points of MDR-TB transmission, with explosive outbreaks, and associated with high mortality. Third, implementation of effective TB infection control can promote awareness of TB, and the adoption of improved practices for the diagnosis and treatment of TB, particularly in the private health sector. Low-cost administrative interventions are feasible and, if implemented, should require minimal resources. Given the evidence summarized in this review, national TB-control programs and public health agencies in LMICs must begin to address nosocomial TB transmission as an integral part of their TB-control efforts. HCWs are essential in the fight against TB, and their health needs to be protected as well as that of patients with TB. With the recent emergence of extensively drug-resistant tuberculosis (XDR-TB), the need to implement infection-control measures has been reemphasized by global agencies such as the WHO and the Stop TB Partnership [78]. Efforts are ongoing to update existing infection-control guidelines in the wake of XDR-TB, and to develop programs that are suitable for resource-limited countries. We strongly support these initiatives and call for more resources and partnerships to tackle the chronically neglected problem of nosocomial TB in low-income countries.

## Supporting Information

Table S1 Strategy Used to Search Pubmed to Identify Studies for This Systematic Review (38 KB DOC)

Table S2 Summary of Non-English Studies That Were Excluded from the Review (Seven Studies where an English Abstract Was Available) (47 KB DOC)

## Abbreviations

CI: confidence interval
HCW: health-care worker
IGRA: interferon-gamma release assay
IRR: incidence rate ratio
LMIC: low- and middle-income country
LTBI: latent TB infection
MDR-TB: multidrug-resistant tuberculosis
NTM: non-tuberculous mycobacteria
OR: odds ratio
PPD: purified protein derivative of tuberculin
TB: tuberculosis
TST: tuberculin skin test
TU: tuberculin unit
WHO: World Health Organization
XDR-TB: extensively drug-resistant tuberculosis
