# Supplementary material for: Tuberculosis among Health-Care Workers in Low- and Middle-Income Countries: A Systematic Review
Source: PLoS Med. 2006 Dec 26;3(12):e494. doi: 10.1371/journal.pmed.0030494 (PMC1716189; doi:10.1371/journal.pmed.0030494)
Supplement: Supplementary file 1 [file pmed.0030494.st001.doc]

**S 1: Strategy used to Search Pubmed to identify studies for this systematic review**

| Search #1 | "Tuberculosis"[MeSH] OR "Mycobacterium tuberculosis"[MeSH] OR "tuberculosis"[word] OR "tuberculous"[tiab] OR "tuberculin"[tiab] |
| --- | --- |
| Search #2 | "Health Personnel"[MeSH] OR "Allied Health personnel"[MeSH] OR "Disease Transmission, Professional-to-Patient"[MeSH] OR "Caregivers"[MeSH] OR "Physicians"[MeSH] OR "Medical Staff, Hospital"[MeSH] OR "Nurses"[MeSH] OR "Nurses' Aides"[MeSH] OR "Nurse Practitioners"[MeSH] OR "Students, Medical"[MeSH] OR "Students, Nursing"[MeSH] OR ("health"[ti] AND "worker"[ti]) OR ("health"[ti] AND "care"[ti]) |
| Search #3: | "Cross Infection"[MeSH] OR ("Disease Transmission"[MeSH] OR "Disease Transmission, Professional-to-Patient"[MeSH] OR "Disease Transmission, Patient-to-Professional"[MeSH]) OR ("Occupational Diseases"[MeSH] OR "Occupational Exposure"[MeSH]) OR "nosocomial"[tiab] OR "nosocomial tuberculosis"[word] OR "occupational tuberculosis"[tiab] OR "hospital exposure"[tiab] OR "occupational hazard"[word] OR ("tuberculosis"[ti] AND "worker"[ti]) OR Transmission [sh] OR Transmission [tiab] |
| Search #4 | #2 AND #3 |
| Search #5 | #1 AND #4 |
