# Supplementary material for: Tuberculosis among Health-Care Workers in Low- and Middle-Income Countries: A Systematic Review
Source: PLoS Med. 2006 Dec 26;3(12):e494. doi: 10.1371/journal.pmed.0030494 (PMC1716189; doi:10.1371/journal.pmed.0030494)
Supplement: Supplementary file 2 [file pmed.0030494.st002.doc]

**S2: Summary of non-English studies that were excluded from the review (7 studies where an English abstract was available)**

| **First Author**  **Year**  **Journal** | **Language**  **(Country)** | **Study type** | **Number of participants** | **Principle findings** |
| --- | --- | --- | --- | --- |
| Aksyutina LP 1998  Problemy Tuberkuleza [59] | Russian  (Russia) | Incidence of TB disease | Unknown | 74 TB cases from 1993-96 |
| Priimak AA  1992  Probl Tuberk [60] | Russian  (Russia) | Incidence of TB disease | Unknown | 293.3 per 100,000 working subjects |
| Ostrosky-Zeichner L  2000  Salud Publica Mex [63] | Spanish  (Mexico) | Prevalence of TB infection  Incidence of TB infection | 1617  231 of 1617 | Prevalence of Tb infection at baseline 39.6%  Incidence of Tb infection 20% |
| El Houssine  2004  Sante [65] | French  (Morocco) | Prevalence of TB infection | 227 | No data |
| Laraqui CH  2004  IJTLD (French) [66] | French  (Morocco) | Incidence of TB disease | Unknown | 130 new cases of tuberculosis notified in 4 years.  Incidence rate in HCWs  85.3/100 000 |
| Kohout J. 1999  Prakticky Lekar. [67] | Czech  (Czech) | Incidence of TB disease | Unknown | 40 cases of TB notified within 20 years – from 1979-1999 |
| Kuklova D,. 1989  Vnitrni lekarstvi. [68] | Czech  (Czech) | Incidence of TB disease | Unknown | No data |
